# Complete Genome Sequences of Two Acetic Acid-Producing *Acetobacter pasteurianus* Strains (Subsp. *ascendens* LMG 1590^T^ and Subsp. *paradoxus* LMG 1591^T^)

**DOI:** 10.3389/fbioe.2017.00033

**Published:** 2017-05-17

**Authors:** Baolei Jia, Byung Hee Chun, Ga Youn Cho, Kyung Hyun Kim, Ji Young Moon, Soo-Hwan Yeo, Che Ok Jeon

**Affiliations:** ^1^Department of Life Science, Chung-Ang University, Seoul, Korea (Republic of); ^2^School of Bioengineering, Qilu University of Technology, Jinan, China; ^3^Department of Agrofood Resources, National Institute of Agricultural Sciences, RDA, Wanju-gun, Korea (Republic of)

**Keywords:** *Acetobacter pasteurianus*, acetic acid bacteria, vinegar, genomic sequence, comparative genomics

## Introduction

Foods and beverages produced by fermentation are essential to human nutrition worldwide and, therefore, have been extensively studied (Sõukand et al., 2015). Vinegar, kombucha beverage, milk kefir, water kefir, and cocoa are the products of acetic acid fermentation (Li et al., 2015). Acetic acid bacteria (AAB) oxidize sugars or ethanol to produce acetic acid, playing an important role in fermentation. AAB have been used historically for various fermentation processes and are Gram-negative obligate aerobic bacteria of the family Acetobacteraceae of *Alphaproteobacteria* (Saichana et al., 2015). Although various bacteria can produce acetic acid, most commercially used bacteria are species of *Acetobacter, Gluconacetobacter*, and *Gluconobacter* (Raspor and Goranovic, 2008). Among these organisms, *Acetobacter* species have attracted much attention in the field of biotechnology because these species are able to tolerate high acetic acid concentrations in the environment (Matsutani et al., 2011).

*Acetobacter pasteurianus*, one species of *Acetobacter*, has been used to brew vinegar worldwide (Gullo et al., 2006). The valuable and useful characteristics of *A. pasteurianus* motivated us to sequence and analyze the full genomes of two type strains of *A. pasteurianus* subspecies: *A. pasteurianus* subsp. *ascendens* LMG 1590^T^ and *A. pasteurianus* subsp. *paradoxus* LMG 1591^T^. Type strain is usually the firstly isolated strain of the species, and exhibits all of the relevant phenotypic and genotypic properties cited in the species circumscriptions. Therefore, the genome sequence of type strain is important to analyze the phenotypic and genotypic characteristics of species (Kim et al., 2014). The genomes of these two strains were compared with other complete genome sequences, and the important proteins involved in acetic acid production are discussed.

## Materials and Methods

### Genomic DNA Isolation

Strains LMG 1590^T^ and LMG 1591^T^, type strains of *A. pasteurianus* subsp. *ascendens* and *A. pasteurianus* subsp. *paradoxus*, respectively, were cultured for 3 days in YPGD media (0.5% yeast extract, 0.5% peptone, 0.5% glycerol, and 0.5% d-glucose) containing 4% (v/v) ethanol. Genomic DNA was extracted using phenol-chloroform extraction and ethanol precipitation. The quality of purified genomic DNA was tested by using NanoDrop 2000 UV–Vis spectrophotometer (Thermo Scientific, MA, USA) and Qubit 2.0 fluorometer (Life Technologies, MA, USA).

### Genome Sequencing and Genome Comparison

The genomes of the two strains were sequenced at Macrogen using two different technologies: Illumina HiSeq and the PacBio single-molecule real-time technique with a 10-kb library (South Korea). *De novo* assembly of the read sequences was carried out using the hierarchical genome assembly process workflow. The annotation of the sequences was carried out using a modified version of the Prokka annotation pipeline, which incorporated Prodigal 2.60, Aragorn, and RNAmmer 1.2 for the prediction of open reading frames, tRNAs, and rRNAs, respectively (Seemann, 2014). Genome comparison among the two strains and other fully sequenced *A. pasteurianus* genomes was carried out by using Mauve software (Darling et al., 2010).

### Direct Link to Deposited Data and Information to Users

The complete genome sequences have been deposited in GenBank under the accession numbers CP015164-CP015167 (*A. pasteurianus* subsp. *ascendens* LMG 1590^T^) and CP015168-CP015171 (*A. pasteurianus* subsp. *paradoxus* LMG 1591^T^) in October 2016. The BioProject designations for strains LMG 1590^T^ and LMG 1591^T^ are PRJNA322127^1^ and PRJNA317328,^2^ respectively. *A. pasteurianus* subsp. *ascendens* LMG 1590^T^ and *A. pasteurianus* subsp. *paradoxus* LMG 1591^T^ are available from the BCCM/LMG Bacteria Collection under accession numbers LMG 1590 and LMG 1591, respectively.

## Interpretation of Data Set

### General Genome Sequence Property

We obtained 218,360 raw reads covering a total of 1,387,777,653 bp with 228× genome coverage for strain LMG 1590^T^ and 146,922 raw reads covering a total of 897,929,341 bp with 124× genome coverage for strain LMG 1591^T^. The complete genome sequence of strain LMG 1590^T^ contained a circular chromosome of 2,859,878 bp with 53.1% G + C content and three circular plasmids with 55.4% G + C content. The genome sequence of LMG 1591^T^ was also assembled into a circular chromosome of 2,810,721 bp with 53.2% G + C content and three circular plasmids with 54.3% G + C content. The general features of the genomes are summarized in Table 1. Briefly, the analyses of the strain LMG 1590^T^ genome identified 2,931 genes. Among them 2,856 genes were annotated as coding DNA sequences (CDSs). A total of 3,163 genes were predicted from the genome of strain 1591^T^ of which 3,088 genes were identified as CDSs. The genome sequences data are available in FASTA, annotated GenBank flat file, graphical, and ASN.1 formats.

**Table 1:** **Genome features of the *Acetobacter pasteurianus* subsp. strains LMG 1590^T^ and LMG 1591^T^**.

Features	LMG 1590^T^	LMG 1591^T^
**Chromosome**		
Contig number	1	1
Size (bp)	2,859,878	2,810,721
G + C (%)	53.1	53.2
Total genes	2,784	2,760
Coding DNA sequence (CDS)	2,709	2,685
tRNA	56	56
rRNA	15	15
Other RNA	4	4
**Plasmid**		
Number	3	3
Size (bp)	49,380/46,811/43,148	259,464/117,661/28,186
G + C (%)	55.4	54.3
Total genes	147	403
CDS	147	403
tRNA	0	0
rRNA	0	0
Other RNA	0	0

### Genome Comparison

**Figure 1:**
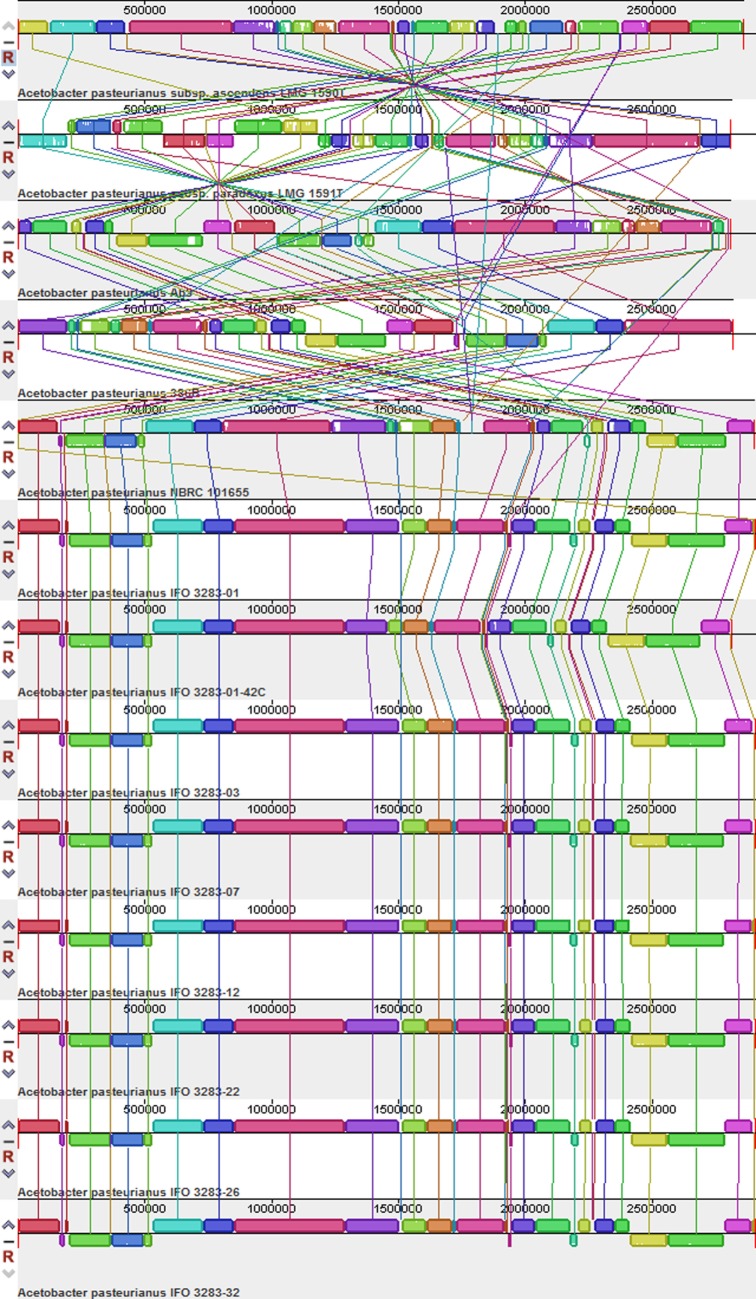
**Global multiple alignments of *Acetobacter pasteurianus* chromosomes**. The 13 genomes were compared to each other using progressive MAUVE with default parameters. Colored blocks outline the genome sequence that aligned to part of another genome and was presumably homologous and internally free from genomic rearrangement (locally collinear blocks). White regions are sequences that were not aligned and probably contained sequence elements specific to a particular genome. Blocks below the center line indicate regions that aligned in the reverse complement (inverse) orientation. The names of the strains are listed at the bottom of the blocks.

To investigate the overall genomic differences between the sequenced *A. pasteurianus* strains, including LMG 1590^T^ and LMG 1591^T^, and the previously sequenced *A. pasteurianus* species, a global alignment of genome sequences from 13 strains was performed using Mauve software (Darling et al., 2010). The results showed that *A. pasteurianus* NBRC 101655, *A. pasteurianus* 386B, and eight *A. pasteurianus* IFO strains were quite similar with respect to genome structure in the chromosome (Figure 1), which was inconsistent with previous reports (Illeghems et al., 2013; Wang et al., 2015). Moreover, rearrangements, deletions, amplifications, and insertions occurred frequently in *A. pasteurianus* Ab3, *A. pasteurianus* subsp. *ascendens* LMG 1590^T^, and *A. pasteurianus* subsp. *paradoxus* LMG 1591^T^ (Figure 1). Similar phenomena were also observed in the plasmids of these strains (Figure S1 in Supplementary Material).

### Acetic Acid-Producing Enzymes

The pyrroloquinoline quinone (PQQ)-alcohol dehydrogenase (ADH) and aldehyde dehydrogenase (ALDH) are responsible for the oxidative metabolism of ethanol to produce acetic acid in AAB. These proteins generally consist of three subunits: a quinohemoprotein catalytic subunit, a triheme cytochrome c subunit, and a third subunit with unknown function. Sequence analysis of the catalytic subunits of the ADHs from strains LMG 1590^T^ and LMG 1591^T^ by a combined transmembrane topology and signal peptide predictor indicated that both of the proteins had a signal peptide and were located in the periplasmic space (Käll et al., 2004). The ADHs were aligned with the well-studied ADH from *Pseudomonas putida* (50% identity to ADHs from LMG 1590^T^ and LMG 1591^T^) (Xia et al., 1996), and the final output was processed using the program ESPript 3.0 (Robert and Gouet, 2014). The results indicated that the PQQ bound to the N-terminal portion, whereas the C-terminal end bound the heme *c* (Figure S2 in Supplementary Material). The ADHs and ALDHs from the 13 strains were further aligned using Clustal Omega (Sievers and Higgins, 2014). Both ADHs and ALDHs from the strains showed high identity (>98%; Tables S1 and S2 in Supplementary Material), although *Acetobacter* species are known to exhibit genetic instability (Azuma et al., 2009). Because ethanol and acetic acid tolerance could be partly attributed to the intrinsic properties of the amino acid sequences of the two proteins and high concentrations of ethanol would not cause mutations in their sequences (Trcek et al., 2006; Zheng et al., 2015), we proposed that the high conservation of the proteins may contribute to the stable industrial performance of *A. pasteurianus*.

In conclusion, the complete genomes of two *A. pasteurianus* subspecies were sequenced and assembled into one chromosome and three plasmids. Comparative genome and sequence analyses showed that rearrangements occurred in the *A. pasteurianus* strains and that the ADHs and ALDHs responsible for acetic acid production were highly conserved in these strains.

## Author Contributions

BC performed the experiments. BJ and CJ analyzed the data and wrote the manuscript. GC, KK, JM, and S-HY helped in data analysis.

## Conflict of Interest Statement

The authors declare that the research was conducted in the absence of any commercial or financial relationships that could be construed as a potential conflict of interest. The reviewer, FF, and handling editor declared their shared affiliation, and the handling editor states that the process nevertheless met the standards of a fair and objective review.

## Footnotes

^1^https://www.ncbi.nlm.nih.gov/bioproject/317327.

^2^https://www.ncbi.nlm.nih.gov/bioproject/?term=PRJNA317328.
