# Supplementary material for: Complete Genome Sequences of Two Acetic Acid-Producing Acetobacter pasteurianus Strains (Subsp. ascendens LMG 1590T and Subsp. paradoxus LMG 1591T)
Source: Front Bioeng Biotechnol. 2017 May 17;5:33. doi: 10.3389/fbioe.2017.00033 (PMC5434130; doi:10.3389/fbioe.2017.00033)
Supplement: Supplementary file 1 [file Data_Sheet_1.PDF]

Supplementary Figure 1

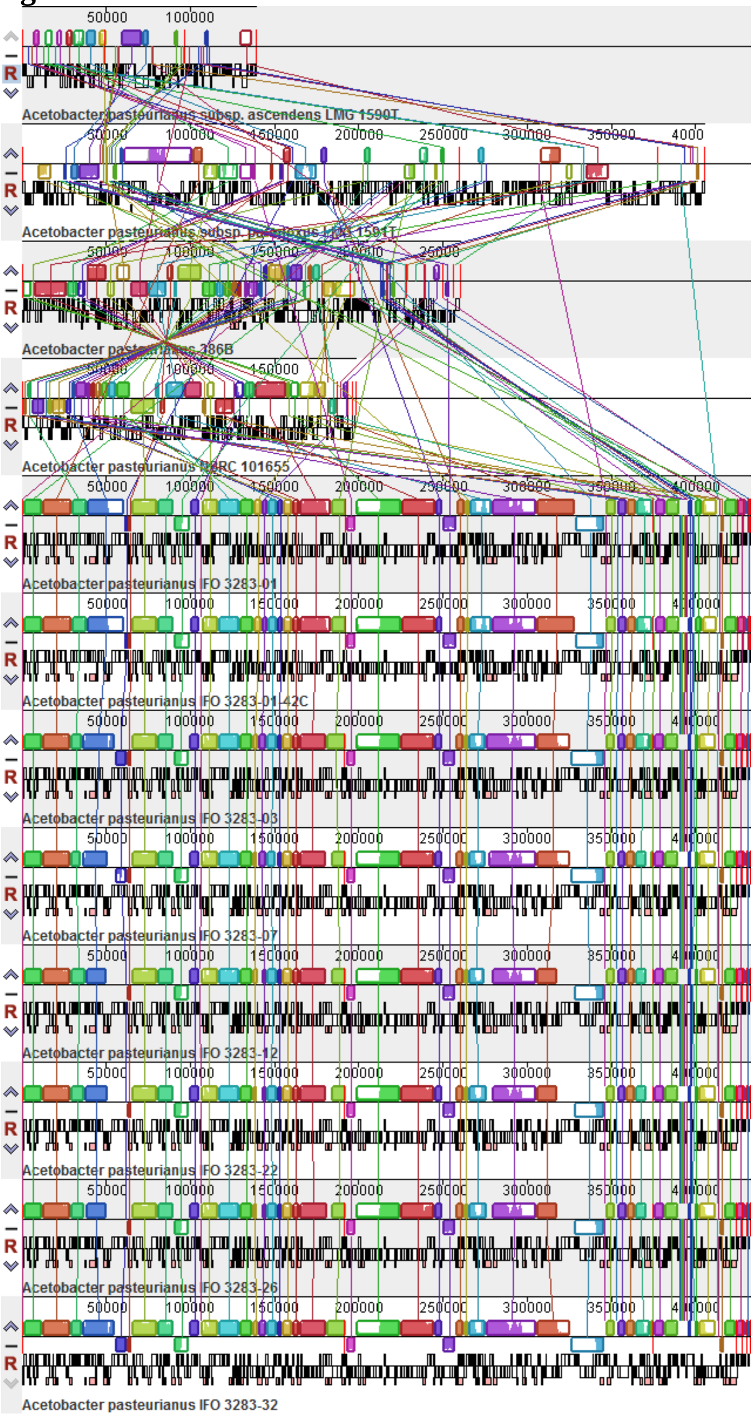

Supplementary Figure 1. Global multiple alignment of plasmids from *Acetobacter pasteurianus*. The plasmids from 13 *A. pasteurianus* were compared to each other using progressive MAUVE with default parameters. Colored blocks outline the genome sequence that aligned to part of another genome and was presumably homologous and internally free from genomic rearrangement (locally colinear blocks [LCBs]). White regions are sequences that were not aligned and probably contained sequence elements specific to a particular genome. Blocks below the center line indicate regions that aligned in the reverse complement (inverse) orientation. The height of the profile within each LCB indicates the average degree of sequence conservation within an aligned region.

Supplementary Figure 2

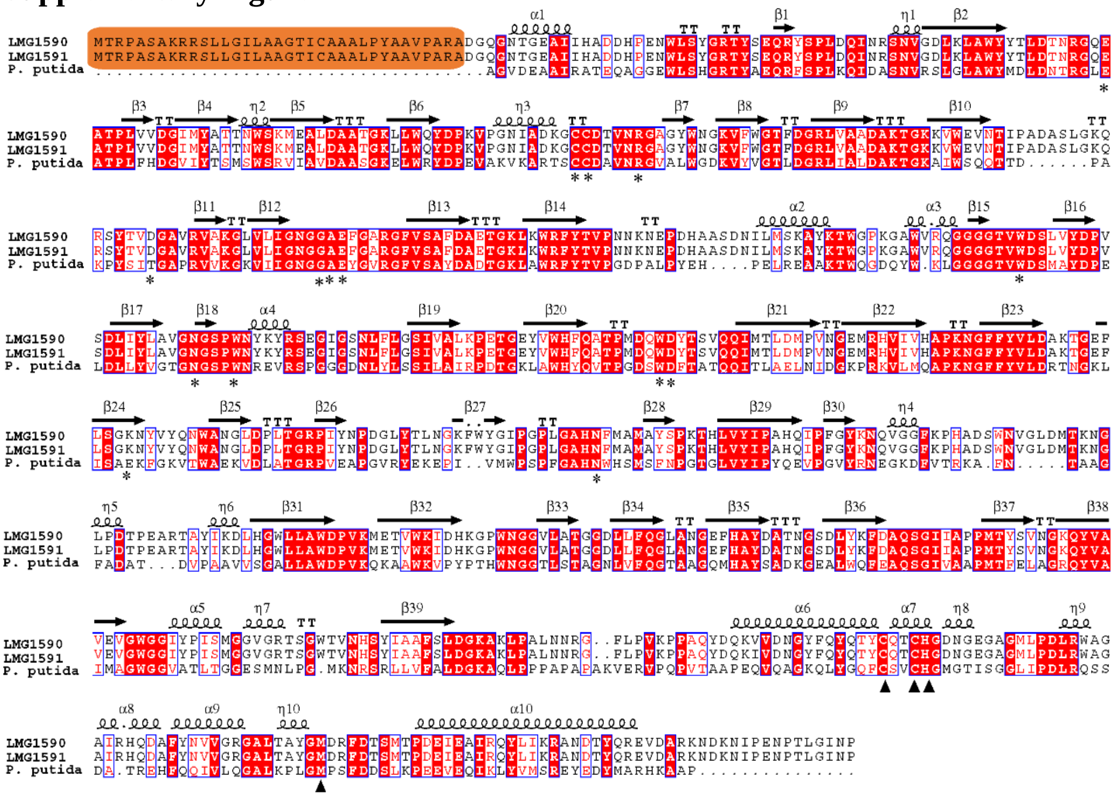

Supplementary Figure 2. Multiple alignments of the alcohol dehydrogenases. Identical residues are in white letters with a red background, similar residues are in red letters with a white background, varied residues are in black letters. The predicted secondary structure is shown at the top of the alignment. α-Helices are represented as helices, β-strands are represented as arrows, β-turns are represented as “TT,” and 310-helices are represented as η. Signal peptides are shown in orange color. Amino acid residues involved in PQQ binding are labeled by stars, and those involved in heme *c* binding are labeled by triangles. Alcohol dehydrogenases are from *P. putida* (WP_058541185.1), *A. pasteurianus* subsp. *ascendens* LMG 1590^T (A4S02_12640), and *A. pasteurianus* subsp. *paradoxus* LMG 1591^T (A4R89_03370).

Supplementary Table 1. Percent identity of the catalytic subunits of alcohol dehydrogenases from *A. pasteurianus*.

Strain name	1	2	3	4	5	6	7	8	9	10	11	12	13
A. pasteurianus subsp. *ascendens* (1)	100.0	99.9	99.7	99.7	99.7	99.7	99.7	99.7	99.7	99.7	99.7	99.7	99.7
A. pasteurianus subsp. *paradoxus* (2)	99.9	100.0	99.6	99.6	99.6	99.6	99.6	99.6	99.6	99.6	99.6	99.6	99.6
A. pasteurianus Ab3 (3)	99.7	99.6	100.0	99.7	99.7	99.7	99.7	99.7	99.7	99.7	99.7	99.7	99.7
A. pasteurianus 386B (4)	99.7	99.6	99.7	100.0	100.0	100.0	100.0	100.0	100.0	100.0	100.0	100.0	100.0
A. pasteurianus NBRC 101655 (5)	99.7	99.6	99.7	100.0	100.0	100.0	100.0	100.0	100.0	100.0	100.0	100.0	100.0
A. pasteurianus IFO 3283-01 (6)	99.7	99.6	99.7	100.0	100.0	100.0	100.0	100.0	100.0	100.0	100.0	100.0	100.0
A. pasteurianus IFO 3283-01-42C (7)	99.7	99.6	99.7	100.0	100.0	100.0	100.0	100.0	100.0	100.0	100.0	100.0	100.0
A. pasteurianus IFO 3283-03 (8)	99.7	99.6	99.7	100.0	100.0	100.0	100.0	100.0	100.0	100.0	100.0	100.0	100.0
A. pasteurianus IFO 3283-07 (9)	99.7	99.6	99.7	100.0	100.0	100.0	100.0	100.0	100.0	100.0	100.0	100.0	100.0
A. pasteurianus IFO 3283-12 (10)	99.7	99.6	99.7	100.0	100.0	100.0	100.0	100.0	100.0	100.0	100.0	100.0	100.0
A. pasteurianus IFO 3283-22 (11)	99.7	99.6	99.7	100.0	100.0	100.0	100.0	100.0	100.0	100.0	100.0	100.0	100.0
A. pasteurianus IFO 3283-26 (12)	99.7	99.6	99.7	100.0	100.0	100.0	100.0	100.0	100.0	100.0	100.0	100.0	100.0
A. pasteurianus IFO 3283-32 (13)	99.7	99.6	99.7	100.0	100.0	100.0	100.0	100.0	100.0	100.0	100.0	100.0	100.0

The accession number of these proteins are as follows: *A. pasteurianus* subsp. *ascendens* PRJNA317327, A4S02_12640; *A. pasteurianus* subsp. *paradoxus* PRJNA317328, A4R89_03370; *A. pasteurianus* Ab3, WP_050818997.1; *A. pasteurianus* 386B, WP_003629834.1; *A. pasteurianus* NBRC 101655, WP_003629834.1; *A. pasteurianus* IFO 3283-01, WP_003629834.1; *A. pasteurianus* IFO 3283-01-42C, WP_003629834.1; *A. pasteurianus* IFO 3283-03, WP_003629834.1; *A. pasteurianus* IFO 3283-07, WP_003629834.1; *A. pasteurianus* IFO 3283-12, WP_003629834.1; *A. pasteurianus* IFO 3283-22, WP_003629834.1; *A. pasteurianus* IFO 3283-26, WP_003629834.1; *A. pasteurianus* IFO 3283-32, WP_003629834.1.

Supplementary Table 2. Percent identity of the catalytic subunits of aldehyde dehydrogenases from *A. pasteurianus*.

Strain name	1	2	3	4	5	6	7	8	9	10	11	12	13
A. pasteurianus subsp. *ascendens* (1)	100.0	99.7	98.7	99.1	99.1	98.6	98.6	98.6	98.6	98.6	98.6	98.6	98.6
A. pasteurianus subsp. *paradoxus* (2)	99.7	100.0	98.7	99.1	99.1	98.6	98.6	98.6	98.6	98.6	98.6	98.6	98.6
A. pasteurianus Ab3 (3)	98.7	98.7	100.0	99.6	99.6	99.6	99.6	99.6	99.6	99.6	99.6	99.6	99.6
A. pasteurianus 386B (4)	99.1	99.1	99.6	100.0	100.0	99.5	99.5	99.5	99.5	99.5	99.5	99.5	99.5
A. pasteurianus NBRC 101655 (5)	99.1	99.1	99.6	100.0	100.0	99.5	99.5	99.5	99.5	99.5	99.5	99.5	99.5
A. pasteurianus IFO 3283-01 (6)	98.6	98.6	99.6	99.5	99.5	100.0	100.0	100.0	100.0	100.0	100.0	100.0	100.0
A. pasteurianus IFO 3283-01-42C (7)	98.6	98.6	99.6	99.5	99.5	100.0	100.0	100.0	100.0	100.0	100.0	100.0	100.0
A. pasteurianus IFO 3283-03 (8)	98.6	98.6	99.6	99.5	99.5	100.0	100.0	100.0	100.0	100.0	100.0	100.0	100.0
A. pasteurianus IFO 3283-07 (9)	98.6	98.6	99.6	99.5	99.5	100.0	100.0	100.0	100.0	100.0	100.0	100.0	100.0
A. pasteurianus IFO 3283-12 (10)	98.6	98.6	99.6	99.5	99.5	100.0	100.0	100.0	100.0	100.0	100.0	100.0	100.0
A. pasteurianus IFO 3283-22 (11)	98.6	98.6	99.6	99.5	99.5	100.0	100.0	100.0	100.0	100.0	100.0	100.0	100.0
A. pasteurianus IFO 3283-26 (12)	98.6	98.6	99.6	99.5	99.5	100.0	100.0	100.0	100.0	100.0	100.0	100.0	100.0
A. pasteurianus IFO 3283-32 (13)	98.6	98.6	99.6	99.5	99.5	100.0	100.0	100.0	100.0	100.0	100.0	100.0	100.0

The accession number of these proteins are as follows: *A. pasteurianus* subsp. *ascendens* PRJNA317327, A4S02_03085; *A. pasteurianus* subsp. *paradoxus* PRJNA317328, A4R89_12235; *A. pasteurianus* Ab3, WP_050819621.1; *A. pasteurianus* 386B, WP_003622886.1; *A. pasteurianus* NBRC 101655, WP_003622886.1; *A. pasteurianus* IFO 3283-01, WP_012812805.1; *A. pasteurianus* IFO 3283-01-42C, WP_012812805.1; *A. pasteurianus* IFO 3283-03, WP_012812805.1; *A. pasteurianus* IFO 3283-07, WP_012812805.1; *A. pasteurianus* IFO 3283-12, WP_012812805.1; *A. pasteurianus* IFO 3283-22, WP_012812805.1; *A. pasteurianus* IFO 3283-26, WP_012812805.1; *A. pasteurianus* IFO 3283-32, WP_012812805.1.
